# Performing Black British memory: Kat François’s spoken-word show *Raising Lazarus* as embodied auto/biography

**DOI:** 10.1080/17449855.2020.1737184

**Published:** 2020-03-19

**Authors:** Julia Novak

**Affiliations:** University of Vienna, Austria

**Keywords:** Poetry performance, spoken word, Black British drama, cultural memory, World War I, autobiography, life writing

## Abstract

Since the 1990s, Black British poets have been at the forefront of developing the “one-person poetry show” or spoken-word play, an apt format for negotiating diasporic history and cultural memory in a public arena. The focus of this article is Kat François’s one-woman show *Raising Lazarus* (2009/2016), which stages the poet’s own quest for information about her Grenadian relative Lazarus François, a World War I soldier. A media-specific analysis explores how François’s text is semantically enriched when translated into a live performance. The authenticity effect typically produced in spoken-word poetry through the unity of author and performer is compounded in *Raising Lazarus* by textual and paratextual keys that frame François’s show as embodied auto/biography. Merging life writing, monodrama, and spoken-word poetry, *Raising Lazarus* reveals the one-person show to be an effective and popular medium for Black British poets to articulate personal experience and negotiate collective identities through performance.

## Introduction

The contemporary British poetry performance circuit functions as an alternative literary scene with its own dedicated agencies, festivals and event series, new event formats, and funding and training schemes, which have created new opportunities for poets across the UK. The spoken-word organizations Apples & Snakes and Jaybird Live Literature, the national poetry slam championships staged by Hammer & Tongue, the Poetry Society’s SLAMbassador scheme and regular Poetry Unplugged nights, and the Performance Poetry module taught by Lucy English at Bath Spa University are just a few examples of this lively scene. Spoken-word poets have returned poetry to its sonic roots by according aesthetic values to the oral performance of their own work and drawing on an accessible idiom. Their success in this has been credited with creating new audiences for the art, particularly young audiences. Indeed, on YouTube, the poems of renowned poet-performers such as Hollie McNish and Benjamin Zephaniah now receive tens or hundreds of thousands of views, figures that are beyond the reach of most print publishers of contemporary poetry.^1^ What is more, British spoken word has long been characterized by a marked prominence of poets of colour, who benefit from the greater accessibility of the performance scene and often draw on a cultural heritage in which oral performance features prominently.^2^ Hence it is unsurprising that Black British poets have also been at the forefront of developing the “one-person poetry show” or “spoken-word play”. From the 1990s onwards – and particularly since the turn of the millennium – the one-person show has become an established spoken-word format in the UK. Renowned exponents such as SuAndi ([1994] 2002; *The Story of M*, first performed 1994), Sissay ([2004] 2017; *Something Dark*, first performed in 2004), and Jonzi (2013; *The Letter*, first performed 2013) have demonstrated how the one-person show can literally lend a voice and visibility to the work of Black British poets, in their customary spoken-word idiom and performance style, and be used to negotiate diasporic history and cultural memory in a public arena.^3^ What is more, many of these one-person poetry shows strategically challenge hegemonic notions of national identity through the lens of autobiography. As the format allows for more extensive treatments of a theme than individual poems, it lends itself well to creatively staging the poets’ personal histories and lived experiences to address broader political questions.

The focus of this article is Kat François’s one-woman show *Raising Lazarus* (2009), which stages the poet’s own quest for information about her Grenadian relative Lazarus François, a World War I soldier. Kat François’s career is a prime example of the opportunities that the vibrant British spoken-word scene has created in recent decades for poets who value oral performance. Raised by her Grenadian mother in the UK, François discovered her passion for performance poetry in her mid-twenties, having previously trained as a dancer. She won the first ever BBC poetry slam in 2004 and the World Slam Championships in 2005. Since then, she has made numerous appearances on radio, television, and at literary festivals, and is today a much sought-after workshop facilitator, frequently taking her work into schools. As a curator and spoken-word compère, she has maintained close ties with London’s Theatre Royal Stratford East, where she developed her first one-woman show *Seven Times Me* in 2007, followed by *Raising Lazarus* in 2009, both under the direction of Dawn Reid. François has since toured worldwide with *Raising Lazarus*, which has seen performances in London, Sydney, Durban, Toronto, and other cities.^4^

Within a few years, François thus managed to forge a remarkable career as a spoken-word poet, earning her living by her craft – a feat for any poet these days. It is worth mentioning that she did so by bypassing traditional poetry publishers. Her first book of poetry, *Rhyme and Reason*, appeared under her own label Zupakat Productions in 2008 and has now reached its third edition. Rather than a reaction to what Danuta Kean and Corinne Fowler (2016, 177) have identified as the institutional racism of the British poetry establishment, partly manifested in an aversion to performance-related work,^5^ self-publication was a conscious choice in François’s case: it was economically more viable for her as she expected to sell most of her books at her live performances, as she explained in an interview with me in London on November 17, 2016. Yet this strategy may well be a reason why François, a highly successful spoken-word poet, has gone largely unnoticed by poetry critics and literature scholars. While poetry is a bi-medial art at base, existing as written and spoken word, literary studies has traditionally focused firmly on the page, paying little attention to the specific aesthetic of poetry’s oral mode, which is key to understanding the work of performance-oriented poets such as François. Habitually, the “sound” of poetry discussed in literary studies is the sound “evoked” on the page rather than that of an actual sonic rendering of a poem, just as the term “voice” is used to denote individual style and implied subjectivity in the abstract, rather than a poet-performer’s vocal production.^6^ As a consequence, poets who eschew the traditional route of publication via an established publisher in favour of performance have a hard time getting noticed by critics and scholars. Given the prominence of Black poets in the British spoken-word scene, it is easy to see how literary criticism’s focus on print impacts disproportionately on the cultural validation and legacy of poets of colour. This neglect designates their creative output (and, in the case of autobiographical spoken-word poetry, the life experience described in the work) as irrelevant, ultimately creating a significant gap in literary and cultural history.

This article examines Kat François’s *Raising Lazarus* as an example of what Deirdre Osborne (2010) has termed the “unique experiential aesthetics” (231) of auto/biographical monodramas by Black British poets. As Osborne argues in her astute analysis of “monodramas” by SuAndi and Lemn Sissay, the extended performance work by spoken-word poets should also be assigned a place in the British dramatic canon. I will use the term “spoken-word play” in the rare instances in which I refer to François’s text in the abstract, and to gesture towards the multiple heritage and transgeneric orientation of François’s work. However, while also drawing on critical studies of monodrama, I will primarily approach François’s show as a specimen of spoken-word poetry, thus keeping its specific provenance and aesthetic in view. For the most part, I will therefore discuss *Raising Lazarus* as a “one-person show”, following current practice within the spoken-word community and focusing on the text’s realization in oral performance.^7^ For this purpose, I will make use of methods developed for a close “reading” of poetry in its oral mode. This article will thus shed light on the ways in which the “experiential aesthetics” of François’s auto/biographical spoken-word play emerges as a specific performance aesthetics. The oral realization of the text enriches it semantically and the text gains further political momentum through the physical presence of the author on stage. Specifically, I will examine how François exploits to the full the authenticity effect typically produced in spoken-word poetry through the unity of author and performer, not just by skilfully manipulating the articulatory parameters of her audiotext and her body behaviour, but also by explicitly framing her performance as an “embodied auto/biography”, through a number of textual and paratextual keys. I will finally consider the effect of these authenticity strategies on the audience, suggesting that they reconfigure *Raising Lazarus* as an open-ended, genuinely dialogic process by which performer and audience jointly “re-member” a chapter of Black British history that is of vital significance for the Caribbean diaspora.

## Authenticity, auto/biography, and the body in spoken word

A concept that occupies a central place in much contemporary spoken-word poetry is that of authenticity. For example, in *The Cultural Politics of Slam Poetry*, Susan Somers-Willett (2009) draws attention to the significance of authenticity for the identity politics of US American poetry slam competitions, where white audiences frequently reward “authentic” performances of Black identity. Her focus on what she sees as the strategic essentialism (with reference to Spivak; see Somers-Willett 2009, 59) of Black identity performances for the purpose of winning slams has incurred criticism from the African American scholar and slam poet Javon Johnson. As Johnson (2017) laments, Somers-Willett “never offers the possibility that the black poets who won, and win, are simply better”, so that “her book at times reads less like a critical inquiry into why black poets are highly rewarded and more like a troubling racial apologia for the lack of white success” (23). Both Somers-Willett’s and Johnson’s positions are founded on aspects of spoken-word poetry that call for forms of critical inquiry alien to traditional poetry scholarship. In the act of speaking, the poet-performer’s identity becomes part of the poem through their physical presence on stage, which in turn creates unique possibilities for poetry that highlights their performed identity. As media philosopher Sybille Krämer (2002, 340) observes, the spoken word seems closely tied up with its speaker, an attribute of the speaker. In poetry performances, this is especially true of first-person texts, where the perceived tie between the actual speaker and the pronoun “I” encourages the audience to attribute an utterance to the poet rather than the intratextual speaker function. In other words, for audiences, poet-performers often seem to project a meta-message of “*I* am saying this, and I really mean it.” Furthermore, in cases when the textual speaker articulates experiences that pertain to, or qualities that characterize, the poet-performer, the performance is easily read as an autobiographical act. In addition to “I really mean it”, a performance may then suggest “I have really experienced this; it is true.”

Much as poets may choose to play with their identity in performance, however, a poem’s quality cannot be reduced to the poet’s identity. Notwithstanding the questions Johnson’s polemic raises about the standards by which to (quantitatively!) judge the artistic quality of poetry performances in slams, his critique of Somers-Willett points to a tendency in academic engagement with poetry performance that is also criticized by Dana Gioia (2004) in *Disappearing Ink*.^8^ In Gioia’s words, “the limited commentary on the new popular poetry” – by which he means oral forms such as performance poetry, spoken word, cowboy poetry, and rap – “has habitually focused on ideological issues, especially in the case of rap, which has been examined almost entirely for its subject matter or sociological significance” (8). What has long been missing from the critical discourse around spoken word is an engagement with the aesthetic dimension of poetry performance.

A productive approach that takes into account both the identity politics and poetics of the spoken word is to focus on the *aesthetic* of authenticity that poet-performers such as Kat François enact. In *Raising Lazarus*, this aesthetic of authenticity functions as a challenge to cultural memory and its lacunae. My claim is that the perceived authenticity of poetry performances is frequently an effect of the chosen performance style, which in François’s case is further enhanced by various acts of framing the poem that position the author as the originator *and subject* of her story. This authenticity effect is fuelled by the audience’s “intense fascination” with the “presence of the authentic subject-body” (Stephenson 2013, 21) that can be turned to political ends. My approach therefore entails a close analysis of performance style^9^ as a central mechanism by which the one-person show can harness auto/biography to stage diasporic identity (Novak 2011).

The term “auto/biography” carries two meanings in this article: it is a shorthand for “autobiography and/or biography” (see Saunders 2010, 6), which is useful when discussing a play like *Raising Lazarus* that deals not only with the poet-performer’s own experiences, but also with those of her relative, whose life she has researched. In this sense, both “biography” and “autobiography” are used here to denote modes of narration rather than literary genres, referring to referential life narratives that take shape in oral performance.^10^ But “auto/biography” also points to the inevitable fusion of the two modes in *any* life-writing project, signalling “the insistence that accounts of other lives influence how we see and understand our own and that our understandings of our own lives will impact upon how we interpret other lives” (Stanley 1994, i–ii). This particular meaning of the term is manifest in *Raising Lazarus* as a structural device. François’s play moves back and forth between two time zones: it intersperses the story of Kat François’s own frustrating research into Lazarus François’s war experiences with imagined episodes of those experiences. François impersonates herself at various stages of her research process, as well as a range of other people – such as the elderly relatives and archivists whom she interviews, her (distant) relative Lazarus, his fiancée Dosue (whom he leaves behind in Grenada), an army recruiter, and a nurse.

On stage, François’s body thus signifies in a number of ways: whenever she represents her earlier self, it is at once a vehicle for the telling of a personal story and that story’s living trace. The “narrating I” of autobiography – to use Sidonie Smith and Julia Watson’s (2010) terminology – emerges via the poet’s body, which is also the material site of the “narrated I’s” experiences (72–73) and now functions to authenticate the poet’s account. Whenever François impersonates other characters, however, switching from one character to another or from her own past or present self into other selves, the various subjects become performatively connected through a shared body. In that sense, the poet-performer’s body constitutes a bridge between past and present, as well as between the locales that *Raising Lazarus* visits. It figures as a truly “diasporic body”, suspended between England and Grenada, between World War I and the early 21st century, all of which have played a role in the formation of the auto/biographical subject. This temporal, spatial, and also figural interconnection brought forth by François’s performance has important epistemological implications, as we shall see.

## Authenticity and performance style

Like other spoken-word plays, *Raising Lazarus* features a blend of poetry and prose passages, of which François said in her interview with me on November 17, 2016:

The two things have developed side by side. I’ve never written a straight play, and I never will. [...] I almost look at it like a musical, you know, when there’s a heightened sense of emotion I do some poetry instead of a song.

This “heightened sense of emotion” becomes apparent in *Raising Lazarus* in the way the words are delivered on stage. The poet-performer’s perceived emotional involvement with the material gives the audience reason to read the poet as embodying the textual speaker, the “I” in the poem, creating the authenticity effect that is typical of spoken-word poetry. This involvement is communicated through audiotext – Charles Bernstein’s (1998) term for “the poet’s acoustic performance” (13) that includes paralinguistic features such as pitch, rhythm, and volume – and through body communication in the forms of posture, gesture, and facial expression. The following analysis will shed light on the connection between performance style, authenticity, and autobiography in spoken-word poetry. The video extract in question is accessible online (see François 2016c).

Towards the beginning of her show, Kat François relates her memories of finding out in 2009 that she is related to Lazarus François, who fought in World War I. In a prose passage, François expresses her surprise and irritation about never having heard of Lazarus from her family in a performance style that can be described as conversational or realist: François faces and addresses her audience directly, in a manner typical of spoken word, and performs her text as though spontaneously telling a story, establishing eye contact, modulating the rhythm and pitch of her speech to suggest excitement, bewilderment, irritation, etc. She also complements her utterances with appropriate gestures and facial expressions. Then a regular beat sets in, and François performs a poem that refers to various incidents of racism she has experienced. The performance style of the prose passage is largely carried over into the poem, though François’s tone becomes somewhat less conversational, her timbre more tense, perhaps even “accusatory”. Prose and poetry are firmly connected by the repetition of “I wish I’d known”, an exclamatory phrase that ends the prose passage and is then used as a refrain at the beginning of each stanza. These repetitions stress the significance François attributes to Lazarus’s story and, through the connection they establish between prose and poetry, frame the poem as part of the *personal* story she tells her audience.

As in any reading of poetry, it is worth paying close attention to the choice and arrangement of words. The spoken word in performance, however, creates meaning on additional levels. Thus, if the speaker is visible to us, their speech ought to be studied in its totality, as “a verbal-paralinguistic-kinesic continuum”, as Fernando Poyatos (1993, 122) insists. And indeed, François achieves a number of effects on stage that cannot be found on the page. Take the following sequence, for example:

I wish I’d known about Lazarus when the National Front marched down my Highstreet. Marching, chanting, shouting, eyes cold, attitude bold, racist leaflets in hand. Shorn hair and red laces, their NF brand. (François 2016c, 01:23–01:37)

**Figure 1.**
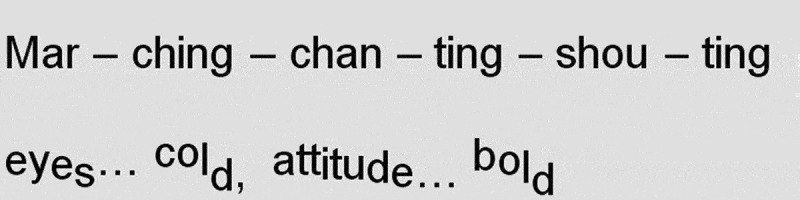
The National Front: verbal transcript indicating pitch movement.

**Figure 2.**
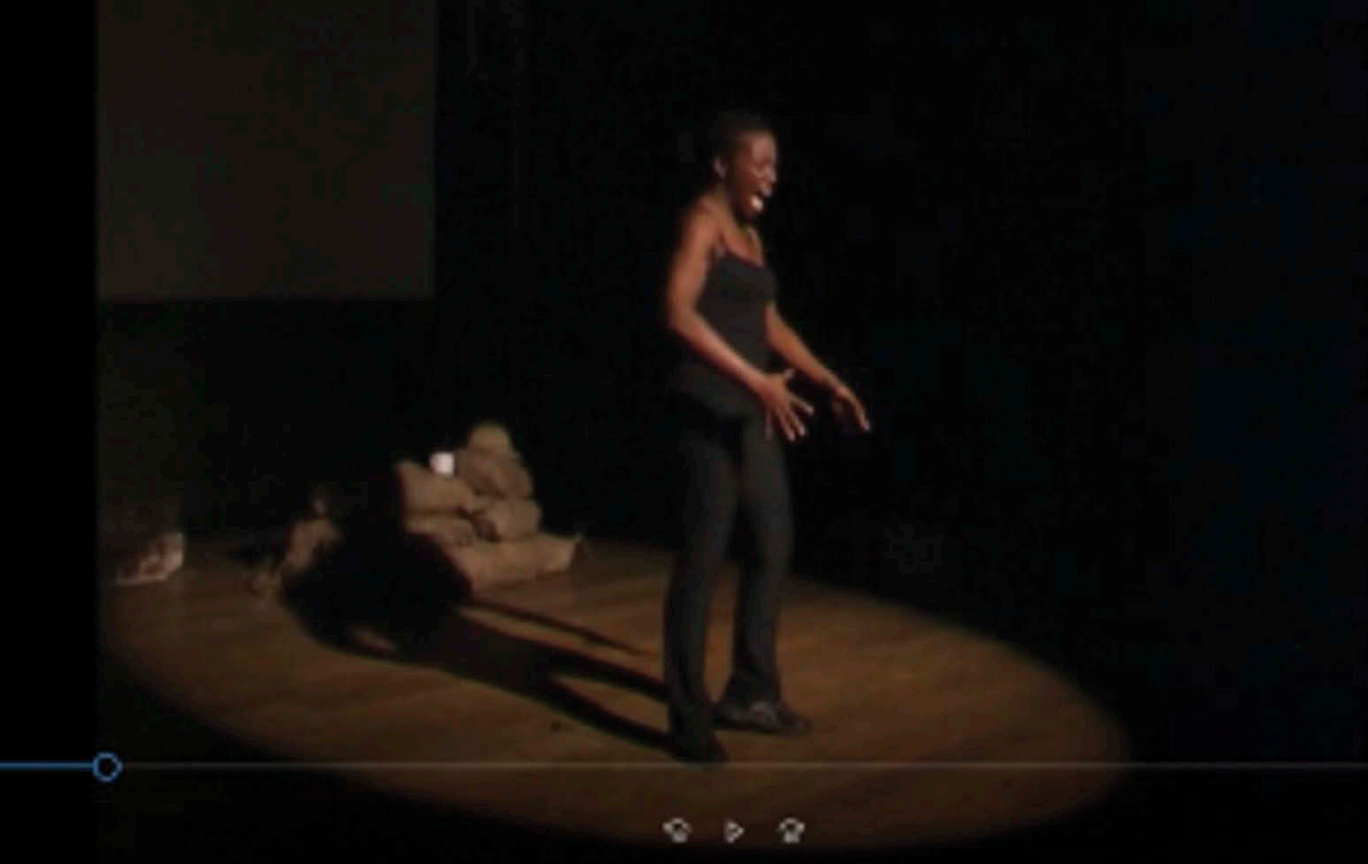
“Eyes … cold” from “I wish I’d known” (François 2016c, 1:31).

“Mar-ching, chan-ting, shou-ting” are uttered monotonously, in a highly regular rhythm. François is acoustically imitating the National Front’s (NF) militaristic marching step here, perhaps even alluding to nationalist activism as monotonous, repetitive, and dull. However, the greater pitch range of “eyes cold, attitude bold” (see Figure 1) and the accompanying distorted face of the poet-performer suggest that the marchers were actually threatening and that François was afraid of them. When uttering “eyes … cold” (ellipses here indicate a pause), François looks down and pushes her hands down, fingers outstretched like claws (see Figure 2). In this manner, her performance indicates that the NF supporters look down on non-white people. It can be read as a physical representation of their aggressive assumption of superiority.

Thus, the greater emotional range resulting from François’s use of pitch,^11^ in conjunction with her facial expressions and gestures, communicates the ambivalent emotions that colour the poet-performer’s account of the NF march. Whether or not François (re-)experiences the terror of her childhood days on stage is beside the point here. Her autobiographical performance is convincingly charged with the outward manifestations of affect that *represent* her emotional response at the time of the NF march – hence the term authenticity *effect*.

Another example of the meaning-making potential of performance is the poet’s rendering of the “black pig” stanza:

I wish I’d known about Lazarus when a …
blond-haired blue-eyed girl in my class called me a …
black pig …
Thought it was funny to laugh at my race,
thought it was funny to curse me in my face,
until I slapped her and put her in her place. (François 2016c, 01:37–01:50)

**Figure 3.**
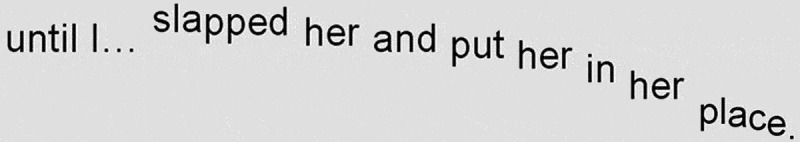
The “black pig” incident: verbal transcript indicating pitch movement.

François pauses before “black pig”, presumably for emphasis, to stress the hurtfulness of that expression, almost as though she was having difficulty repeating it aloud. By contrast, “slapped her and put her in her place” is uttered quickly and fluently after François has performed the corresponding slapping gesture. The steady pitch fall from “slapped” onwards not only communicates finality (in the sense of, “this insolent girl was taken care of”), but also de-emphasizes François’s utterance (see Figure 3).

This “deactivation”, as Theo Van Leeuwen (1999, 111) terms the falling pitch, makes it sound as though slapping the girl were the most ordinary thing in the world – an implicit suggestion that the audience meets with laughter. Again, François’s audiotext communicates additional facets of meaning here that cannot be gleaned from the page. More importantly, where the written text might have failed to create sympathy for the autobiographical subject (a girl who resorts to violence to settle her disputes), the humour in the poet’s performance draws the audience in. The ensuing laughter also highlights the (temporary) collectivity into which the audience enters through this act of witnessing.

**Figure 4.**
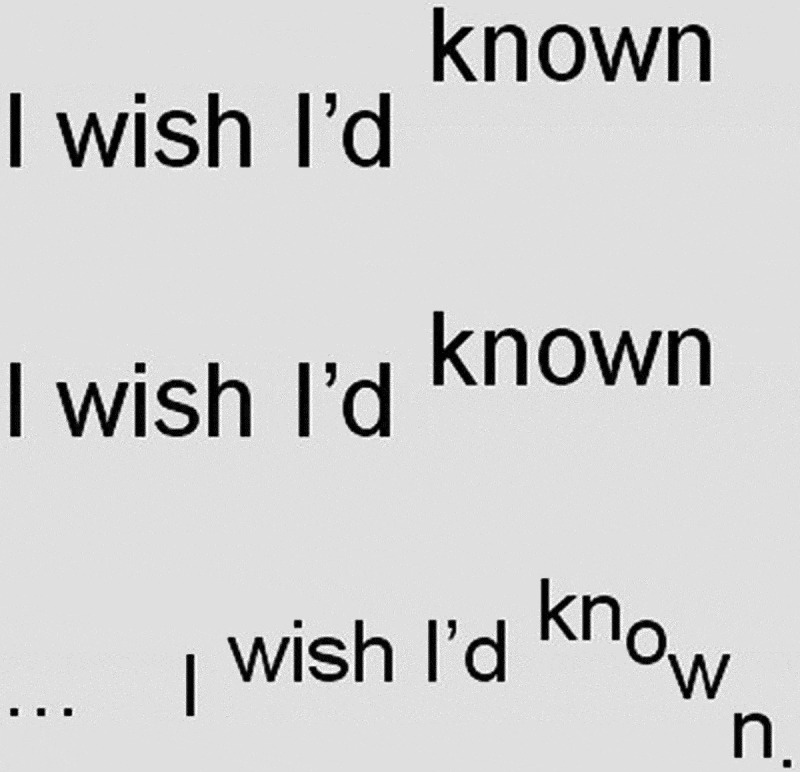
“I wish I’d known”; verbal transcript indicating pitch movement and volume.

François ends the poem on “I wish I’d known” (02:37–02:43), which is repeated three times. The first two repetitions have very similar pitch curves and the same tense, accusatory timbre that she uses in the rest of the poem, suggesting annoyance at not having learned about Lazarus earlier (Figure 4). The third repetition is marked by a softer timbre and lower volume, coinciding with a shrug of the shoulders that creates an overall impression of sadness and regret in the realization that knowing about her ancestor’s contribution to the British war effort would have had a vital impact on her cultural sense of self and strengthened her in her encounters with racism. This insight, foundational to François’s show, is expressed most clearly in the penultimate stanza of “I Wish I’d Known”:

I wish I’d known about Lazarus when old white people would say,
“Why don’t you go back to where you come from.”
Or “My father and grandfather didn’t fight in the war
for this country to be taken over by your sort.”
(François 2016c, 02:10–02:20)

At this point it is worth reflecting on *Raising Lazarus* through the lens of autobiography theory to gauge the cultural work François performs by linking her own and her ancestor’s life narratives on stage. Narrative theories of identity suggest that life narratives bring identity into being rather than representing what is already there. Autobiographical narratives are “a mode of phenomenological and cognitive self-experience”, John Paul Eakin (1999) asserts, “while the self – the self of autobiographical discourse – does not necessarily precede its constitution in narrative” (100). In this sense, François’s self-narrative in *Raising Lazarus* amounts to a claim to identity, a construction of the autobiographical subject Kat François. Critics of autobiographical monodrama have pointed out that such narrative acts of identification assume particular poignancy when they occur on stage – as “a way to speak oneself into being”, as Jenn Stephenson (2013, 10) notes, in which performance figures as “a strategy to grant self-possessive agency to the performing subject and thereby avert objectification and marginalization” (10). Stephenson’s conception of autobiographical performance already implies that the stage constitutes a crucial site of self-construction particularly for identities under pressure from objectification and marginalization. It functions as a space where marginalized subjects, proclaiming themselves in what Deirdre Osborne (2013) terms a “feat of resistant orality” (56), can make themselves seen and their voices heard.

But *Raising Lazarus* enacts another central tenet of life-writing theory: that the story of our individuation is at once the story of our relation to others – a notion also invoked by the slash in auto/biography. In other words, if “*all* identity is relational”, as Eakin (1998) claims, the stories of others are implicated in the narrative formation of the self: “narrative is a – if not the – principal mode in which relational identity is formed and transacted” (63; original emphasis). *Raising Lazarus* dramatizes François’s research of her ancestor’s story as a quest for self-knowledge. In “I Wish I’d Known”, this vital connection between the two is made explicit. In retrospect, it figures as a lack, a missed chance for young Kat to draw on the story of her ancestor in order to bolster her sense of self and claim her place in the world. Her poem suggests that knowing about Lazarus would have strengthened her in her encounters with ethnic nationalism and made her more self-assured. As such, *Raising Lazarus* does not just destabilize the autonomous self of traditional western autobiography – a typical effect of life narratives with a strong emphasis on relationality, as well as of postcolonial life writing in particular (see Eakin 1999, 69; Moore-Gilbert 2009, xviii–xxi; Smith and Watson 2010, 218). It insists, at the same time, and paradoxically, on the stability that relationality can afford the individual as an opportunity for identification.

In both respects – the performative narration of the self and its integration of someone else’s story – performance style is key in *Raising Lazarus*. The emotional and semantic nuances of François’s performance turn the show into a very personal, almost intimate, act of poetic storytelling. They set in motion the workings of affect contagion as “a corporeally based form of mimetic communication” (Gibbs 2011, 259). The co-presence of audience and poet-performer as well as the direct form of address allow for a more immediate “reading” of emotions. The professed sincerity (“I really mean it”) lends François’s story additional persuasive force, authenticating it as autobiography (“I have really experienced this, it is true”) and, by implication, marking Lazarus’s story as a significant element in her self-account.

## Framing authenticity: Textual, paratextual, and co-textual keys

The authenticity effect produced by François’s skilful manipulation of audiotext and body communication is compounded in *Raising Lazarus* by various keys – textual, paratextual, and co-textual – that frame the show as auto/biography. The theatre’s promotional flyer advertises *Raising Lazarus* as charting the “true story” of British-born Kat François searching for information about her relative Lazarus François. The characters’ names in the play establish a connection with its author. Kat François appears as the *author* of her “true story”, as its teller, or performer, and also as its *subject*. These textual and paratextual cues thus lead the audience to enter what Philippe Lejeune (1975) termed the “autobiographical pact”, which specifies the unity of a text’s author, speaker, and subject, and the idea that the author will tell her story truthfully, or at least attempt to do so (26). Arguably, the criterion of truthfulness also extends to François’s performance of her own grandmother, whose utterances and manner of speech she can re-enact from memory.

**Figure 5.**
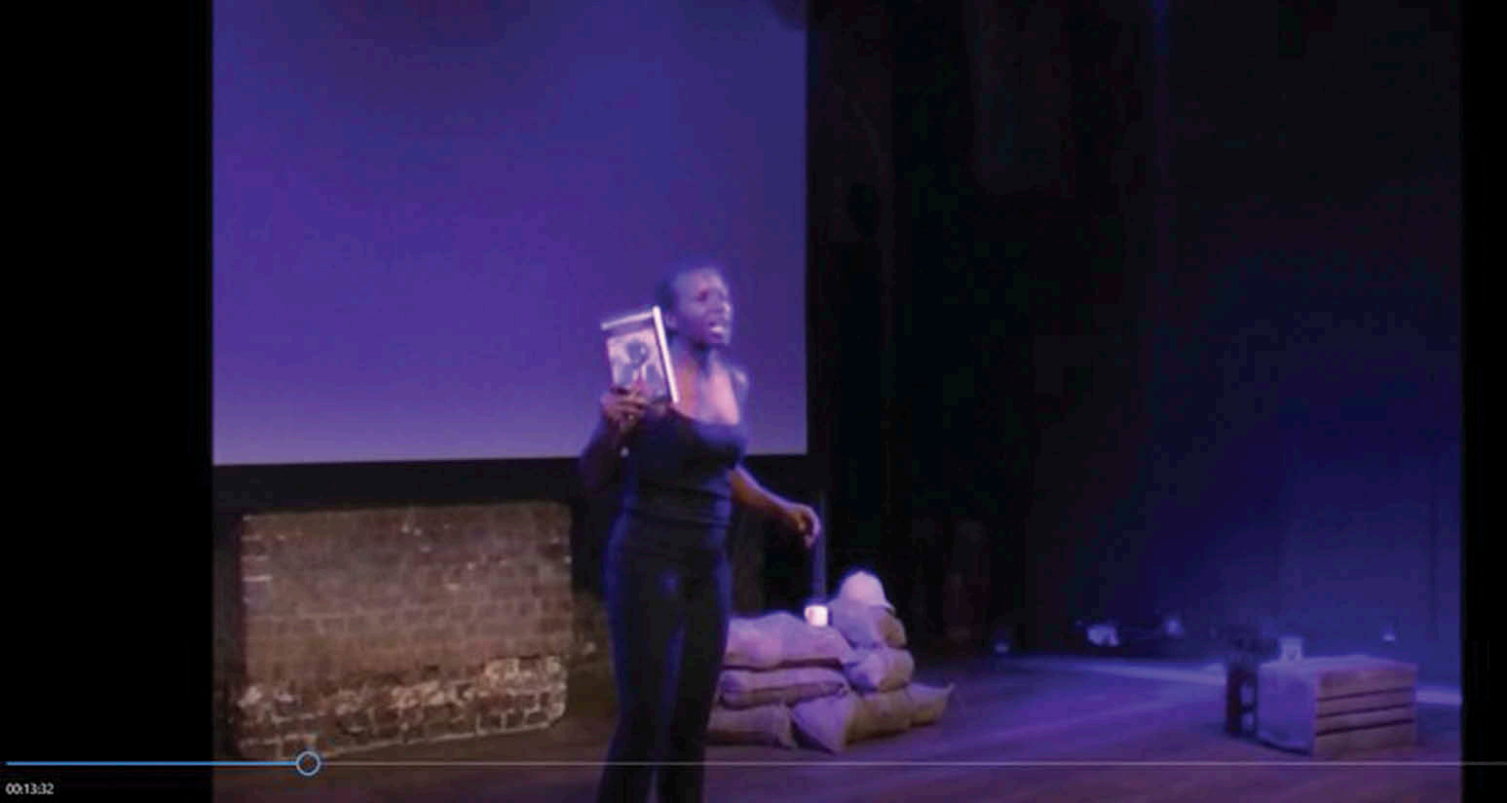
François holding up Glenford Howe’s *Race, War and Nationalism: A Social History of West Indians in the First World War* (François 2016d, 13:33).

However, François requires additional strategies to authenticate the story of Lazarus’s wartime experiences, as her great-grandfather’s cousin lived and died before she was born. For this purpose, she uses a number of props as well as images and video footage that are projected onto a screen behind her. When she recounts how she began her research, she holds up several of her sources, such as Glenford Howe’s (2002) *Race, War and Nationalism: A Social History of West Indians in the First World War* (Figure 5). I would argue that in naming and showing her sources, the poet encourages her audience to discover them for themselves and learn more about Caribbean history. But apart from the educative function of these props, the books are also used as evidence that François *did* consult scholarly sources, that her account is based in research and is thus verifiable.

**Figure 6.**
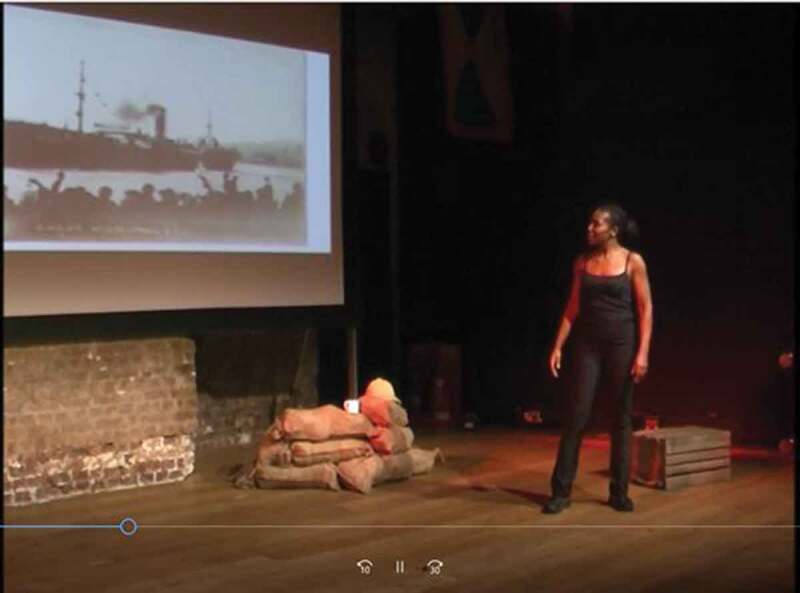
Troop ship SS *Verdala*, 1915 (François 2016d, 18:03).

Two examples of the images François uses are scans of war diaries of the British West Indies Regiment that she examined in the National Archives, and of the troop ship SS *Verdala* (Figure 6), which shipped the first contingent of soldiers from Grenada to England in August 1915. An example of the video footage included in *Raising Lazarus* is of soldiers of the British West Indies Regiment marching in the Middle East. Like the material props and projected photographs, it functions to counteract the fictionalization that must inevitably take place in François’s impersonations of Lazarus, about whom little documentation is available other than a handful of official records, one photograph, and a couple of clues to his character that reached the poet by way of family lore. She thus appropriates the evidence and sources she has collected about the British West Indies Regiment to add authenticity to her fictional story of her relative and thereby ground it in historical fact.

**Figure 7.**
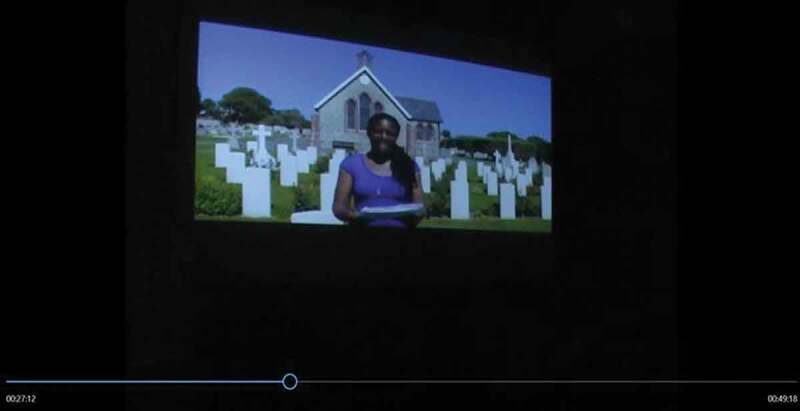
François at Seaford cemetery: “Here They Lay” (François 2016b, 00:40).

**Figure 8.**
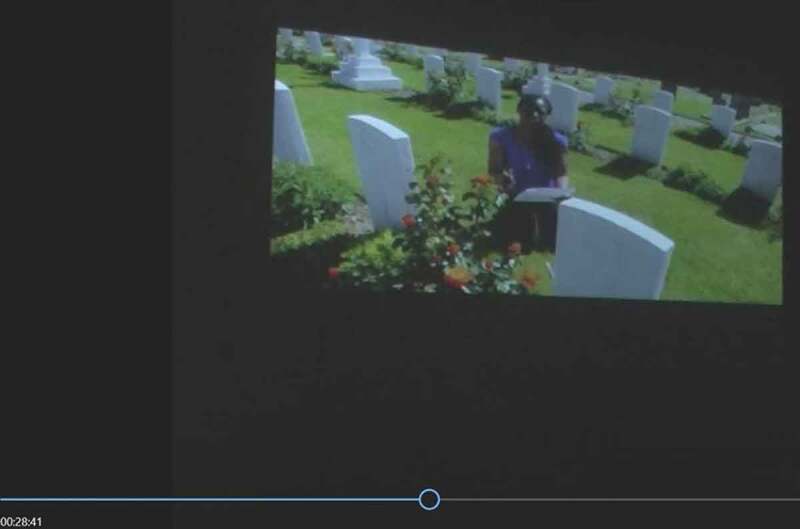
François at Seaford cemetery: “Here They Lay” (François 2016b, 00:40).

Another example of the use of video footage as an authentication strategy occurs when the stage darkens and footage is shown of Kat François reciting her poem “Here They Lay” (François 2016b) at Seaford cemetery, where 19 soldiers of the British West Indies Regiment lie buried (Lazarus is not one of them – he died in dubious circumstances on a ship). The poem in the video begins with an establishing shot of Seaford cemetery (Figure 7) and concludes with François kneeling before the grave of one of the fallen soldiers, speaking up into the camera (Figure 8). The poem is thus visually framed as a critical comment on British history and its forgotten colonial war dead, and as an expression of François’s personal emotional involvement as she kneels, almost piously, among the graves. Its remediation in the show serves the purpose of authenticating both François’s account of her quest for Lazarus, proving that she really was at Seaford Cemetery, and her account of the fallen Caribbean soldiers, whose graves are shown in the footage.

A generic *lieu de mémoire*, the cemetery assumes an additional function in François’s show. It points up the way in which the close connection between autobiography and biography opens out into the domain of cultural memory. As much as individual identity depends on remembering our own past, it draws on collective memories of the history of communities with which we “identify” (Assmann 1992; Erll 2011). In that sense, Lazarus François represents more than a distant relative of Kat’s, more than a point of personal and familial reference: he is introduced as a figure of shared identification that tests the boundaries of the national imaginary. Through the social levelling of soldiers in death, visually represented by the even rows of tombstones typically found at military cemeteries, the fallen soldier has long served as a focal point of nationalist sentiment, across divisions of social class. This “cult of the fallen soldier”, as John Gillis (1994, 13) terms it, is mobilized in *Raising Lazarus* to affirm the rightful place of British Caribbean subjects in the British nation on the grounds of a shared history of warfare and grief. François seems to suggest that if the nation is a product of human invention and based on acts of imagining, as Benedict Anderson (1991) has proposed, its boundaries can be shifted: biography can be employed to integrate Black British experience within the national imaginary and thus rob white nationalists of one of their favourite arguments on which their exclusionary practices are founded. François’s recourse to her ancestor’s story is thus distinctly forward-looking, confirming Stuart Hall’s claim that although identities may ostensibly revolve around a historical past, they are really about “questions of using the resources of history, language and culture in the process of becoming rather than being” (1996, 4). I would argue that Kat François’s show is no less persuasive for the fact that she makes the tenuousness of her knowledge of Lazarus François explicit by dramatizing the numerous difficulties and frustrations of her research process. If anything, it confirms the Caribbean contribution to World War I as a blind spot of British history and national consciousness. As such, *Raising Lazarus* can be seen as part of a larger revisionist project that includes factual and fictional accounts. It performs similar work as Stephen Bourne’s (2014) *Black Poppies: Britain’s Black Community and the Great War*, John MacLaverty’s (2008) *Walter Tull: Forgotten Hero*, David Olusoga’s (2014) TV documentary *The World’s War: Forgotten Soldiers of Empire*, or Andrea Levy’s (2014) short story “Uriah’s War”, all of which challenge the white-washed master narrative about World War I and harness the power of individual stories to convey the bigger picture (see Howe 2002).

## *Raising Lazarus* as an open-ended, dialogic process

The meta-biographical level of *Raising Lazarus* presents Lazarus François’s life narrative – and, by extension, that of Kat François – as unfinished and open to revision. At this point, it is worth reflecting once more on the mediality of François’s text, which challenges traditional conceptions of the literary. When François composed *Raising Lazarus*, she developed it as a stage piece. She wrote the words down to remember them, but this was done as a way of storing rather than publishing the text – *Raising Lazarus* first met a public *in performance*, in 2009. It was not until 2016 that François published the script in her own imprint, Zupakat Publications (François 2016a), by which time the text had undergone several changes. In October 2016, the book was for sale after the performance I attended. It does not represent one particular performance. The exact wording of François’s show, including the poems, varies noticeably from one performance to the next, which also upsets traditional notions of “the poem” or “the play” as literary artefact. Rather, book and performance must be considered in parallel as complementary ways of publishing *Raising Lazarus*, neither of which can claim the status of a stable, original version. *Raising Lazarus* is, and has consistently been, in progress: a text in flux.

François solemnly introduces her show’s final poem with the words “I will continue to look, continue to search, continue to raise Lazarus.” This programmatic statement is borne out by the numerous transformations the show has undergone since its conception, often in step with the poet’s ongoing research into her relative’s life and Caribbean involvement in World War I, as her manager Robert Covell explained in an email interview with me on May 4, 2017. On the point whether Lazarus received any medals, for instance, the book just notes that “he doesn’t have any medals, not even one, never mind” (François 2016a, 20). François later found out that Lazarus was entitled to two medals, something she then mentioned in her performance in October 2016. The year 2016 in particular, as the centenary of the Battle of the Somme, saw the publication of many relevant sources and hence brought about a number of new additions to the show, such as the original footage of the marching soldiers in the Middle East, which forms the background to the poem “Black Soldiers Lament”.

This “openness” of *Raising Lazarus* is further manifest in the question-and-answer (Q&A) session after François’s performance, which forms a set part of her show. “The Q&A was important for me because I think it’s [...] an unexplored subject”, François explained in her interview with me on November 17, 2016: “We just found the audience always wanted to talk after the show, so we thought, ok, why don’t we make that part of the show.” Arguably, it is both François’s realist performance style – the emotional urgency she projects on stage – and the auto/biographical framing of her quest for Lazarus as a personal story which render her approachable and encourage the audience to ask questions. François also finds the dialogue with her audience beneficial, as audience members sometimes give her clues to relevant sources. On the night I attended her performance, a woman raised her hand to say that she was part of a Caribbean history research group with access to material such as birth, marriage, and death records throughout the Caribbean, suggesting they might be able to fill in some of the gaps in François’s research. After the performance, I asked François whether the show would be different again in a year’s time, and she replied: “It will be. I already know.”

## Conclusion

In so far as François addresses the experience of colonial subjects and the (residual) affective structure of colonial hierarchies more broadly, *Raising Lazarus* can be read in the light of autobiographical accounts such as *The Interesting Narrative of the Life of Olaudah Equiano, or Gustavus Vassa, the African* (Equiano 1789). It follows a tradition of testimonies in colonial and postcolonial life writing that represent the experiences of a community, embracing what Gillian Whitlock (2015) identified as an “ethics of witnessing and sympathetic interestedness” which, by nature of appealing to an addressee, is “dynamic and interactive” (7–8). As such, *Raising Lazarus* is intended as an intervention in Britain’s cultural memory and national consciousness by way of auto/biography. At times, it may convey a simplistic view of heroic warfare – after all, *Raising Lazarus* is neither pacifist in spirit nor does it question the role of the UK in 20th-century world politics. In terms of its critical take on the national imaginary, however, its testimonial remit would seem particularly relevant at a time when the British government is once again policing the borders of the nation along ethnic lines (exemplified in the so-called “Windrush scandal” of 2018, in the course of which it emerged that documents of Black British lives had literally been obliterated [see e.g. Gentleman 2018]) and has exited from the European Union, with an ensuing ethno-nationalistic surge among some groups of the population (Booth 2019; Valluvan 2019). Against these tendencies, François mounts her spoken-word show as a meta-biographical, postcolonial critique of Britain’s failure to commemorate its Black soldiers like its white ones. And indeed, François’s claim in her interview with me on November 17, 2016, that “people are ready to hear a different side of history” is reinforced by the Imperial War Museum’s invitation to perform *Raising Lazarus* as part of their commemorative event “The Night Before the Somme” on June 30, 2016, pointing to a change of awareness on an institutional level.

Although François’s account of the Black contribution to World War I must be considered testimony-by-proxy, her embodiment of Lazarus François works to re-present her ancestor on stage with all the immediacy that the spoken word affords. The feat François accomplishes in performance, then, is her creation of “knowable forms of diasporic identity”, which Elspeth Tilley (2012, 311) considers one of the strengths of diasporic monodrama. The diasporic subject first needs to be recognized as a subject before it can be committed to cultural memory and absorbed in the national imaginary, even if its form remains provisional. But as a specimen of spoken-word poetry, *Raising Lazarus* does more than that: François harnesses the authenticity effect typical of the spoken word to forge an indissoluble connection between text and poet-performer, framing her show as an embodied auto/biography and, as such, an ongoing process of (self-)discovery, which the audience is encouraged to share. Only by paying close attention to the nuances of meaning in her oral delivery can audience and researcher fully understand her innovative use of auto/biography. Merging life writing, monodrama, and the conventions of spoken-word poetry, *Raising Lazarus* thus reveals the one-person spoken-word show to be an effective and popular medium for Black British poets to articulate personal experience and negotiate collective identities through performance.

## Notes

1. The immense popularity of some spoken-word poets has recently given rise to a controversy in the poetry world, when Rebecca Watts attacked Kate Tempest, Rupi Kaur, and Hollie McNish for what she perceives as their lack of craft (see Flood and Cain 2018).

2. Opportunities for black poet-performers have also been created by major spoken-word organizations such as Apples & Snakes, Renaissance One, Tilt, or Spread the Word, which have long demonstrated a commitment to diversity. A recent example is the Breaking Ground project launched by Speaking Volumes Live Literature Productions in 2017 to celebrate writers of colour.

3. Further examples are Valerie Mason-John’s *Brown Girl in the Ring* (1998), Roger Robinson’s *Shadow Boxer* (2001), Malika Booker’s *Unplanned* (2007), Nick Makoha’s *My Father and Other Superheroes* (2009), Inua Ellams’s *The 14th Tale* (2009), Cheryl Martin’s *Alaska* (2016), and Yomi Sode’s *COAT* (2017).

4. I saw *Raising Lazarus* (2016b, 2016c) on October 19, 2016 at the Roundhouse Theatre in London and got permission to record it. My observations and analyses refer to this particular performance.

5. While Bernardine Evaristo laments the fact that “performance poetry has become synonymous with poetry from Black writers” (quoted in Keane 2004, 16), poet and avid performer Patience Agbabi points out that a central cliché attached to performance poets is that their performances are “just masking bad writing” (quoted in Novak 2011, 31). A bias against performance-related work must thus be seen as also ethnically inflected.

6. Introductions to the study of poetry – such as Perrine (1982); Matterson and Jones (2000); Eagleton (2007); Bode (2001); Furniss and Bath (1996) – are a revealing genre in this respect, treating, as they do, suitable methods for the study of poetry. For a more extensive critique of the omission of poetry’s oral mode and the deployment of quasi-acoustic terminology in primers, see Novak (2011, 52–56).

7. The spoken-word community evidently conceives of this extended form of performance work as a genre of spoken-word poetry rather than associating it with theatre or drama. For instance, in a 2014 interview, Patience Agbabi remarked that the popularity of the “one-person show” – rather than “play” or “drama” – has created new opportunities for poets to attract public funding (see Novak and Fischer 2016, 360). Osborne (2010), too, stresses the distinct spoken-word element: “The use of ‘I’ [...] is also further shaped and constrained by the poetic systems to which the writers adhere as poets, first and foremost, rather than as dramatists” (232).

8. Poetry slams are frequently judged by randomly selected audience members who raise score cards.

9. In this, I will mostly draw on the methodology for performance analysis developed in Novak (2011).

10. Life-writing scholars (Moore-Gilbert 2009, xi–xvii; Whitlock 2015, 3) frequently point to the Eurocentric history and bias of “autobiography” as a genre term and eschew the label (except in adjectival form) and its perceived cultural baggage for the more inclusive “life writing”. I use it here to expand the traditional concept of autobiography to include stage performance, and avoid neologisms such as “life-speaking” or auto/biophony.

11. Van Leeuwen argues convincingly that an increase in pitch range signals the venting of strong feelings such as “excitement or shock, [...] grief or joy” (1999, 106). A detailed methodology for studying poetry performances can be found in Novak (2011).
